# Teaching writing in primary education (grades 1–6) in Australia: a national survey

**DOI:** 10.1007/s11145-022-10294-2

**Published:** 2022-05-05

**Authors:** Anabela de Abreu Malpique, Debora Valcan, Deborah Pino-Pasternak, Susan Ledger

**Affiliations:** 1 School of Education, Edith Cowan University, 2 Bradford St, Mount Lawley, WA 6050 Australia; 2 Faculdade de Psicologia, CICPSI, Alameda da Universidade, 1649-013 Lisbon, Portugal; 3 Murdoch University, Perth, Australia; 4 University of Canberra, Canberra, Australia; 5 The University of Newcastle, Callaghan, Australia

**Keywords:** Writing instruction, Teacher-efficacy, Teacher preparation, Primary grades

## Abstract

Providing adequate writing instruction and practice in schools is an essential cornerstone of writing development and it affords a diagnostic approach for teachers. But what writing instruction is being practiced in Australian primary schools? The aim of this study was to survey a sample of teachers (n = 310) about their instructional practices for writing and their preparation and self-efficacy to teach writing. The majority of the teachers surveyed indicated they allocated on average less than three hours per week for writing practice in their classrooms, with findings further showing a large variability in the frequency of writing practice ranging from 15 min to 7.5 h per week. Findings suggested an emphasis placed on teaching foundational skills, such as spelling, over the teaching of process skills, such as planning and revising. Results further indicated that less emphasis is placed on teaching handwriting and typing. The majority of participating teachers reported implementing only six of the 20 different instructional practices included in the survey on a weekly basis, with school-home strategies being the least frequently reported strategies to foster students’ writing development. Most teachers expressed positive beliefs about their preparation and self-efficacy for teaching writing. Results from multiple regression analysis showed that preparation and self-efficacy for teaching writing significantly and statistically accounted for variability in using evidence-based practices, teaching foundational skills, and teaching process skills. However, only self-efficacy made a statically significant contribution to predicting strategies to extend writing to the home environment. Implications for teaching and recommendations for research are provided.

## Introduction

Skilled writing is a core requirement for success in schools and beyond and a key competence for lifelong learning (UNESCO, 2017). Concerns about students’ abilities to develop strong writing skills have been reported across continents and languages of instruction (e.g., Banales et al., 2020; Cutler & Graham, 2008; Veiga-Simao et al., 2016). National standardised tests assessing writing in the United States, for example, showed that three quarters of students write at or below basic proficiency (National Centre for Educational Statistics, 2012). In Australia, national results showed a significant decline in students’ writing performance on the National Assessment Program—Literacy and Numeracy (NAPLAN) in the nine years of the test (Australian Curriculum, Assessment and Reporting Authority [ACARA], 2021; Thomas, 2020).

Learning how to write is a challenging developmental process that is typically taught in schools. According to the Writer(s)-within-Community model (WWC) (Graham, 2018), effective writing development is both shaped and constrained by the context in which it takes place and by the writer’s individual cognitive resources and capabilities. As such, examining the nature of writing instruction in different educational contexts and across schooling is paramount. In the last 30 years, research in various countries across the globe has reported concerns in writing instruction associated with teachers’ preparation to teach writing and the nature and frequency of writing practices and instruction. In Australia, however, national surveys set to gain insights into teachers’ practices for writing instruction have not yet been conducted. The present investigation addresses this gap by surveying Australian primary teachers across states and territories on multiple aspects of writing instruction. If the aim is to enhance pedagogies that support writing development, we argue that a necessary first step is to understand how teachers are currently teaching writing and what opportunities are given to students for writing practice.

### Global picture of writing instruction in primary education

The relevancy of studying writing instruction in individual countries is reflected in the WWC model (Graham, 2018), which postulates the importance of examining individual writers’ factors and macro-contextual factors explaining writing development. Based on sociocultural and cognitive perspectives explaining writing development, the WWC model posits that “writing is a social activity, situated within specific contexts” of learning (Graham, 2018, p. 259) and involves multiple participants. Individual writers are thus part of writing communities, including home and school, and teachers and family members may act as writing mentors who shape and support individual writers’ capacity to communicate ideas into written language. Moreover, teachers’ variables, including teachers’ preparation, knowledge to teach writing, and self-efficacy beliefs to teach writing, are likely affected by each country’s social, political, cultural, and historical factors (Graham et al., 2021; Graham et al., 2021).

Since a key factor explaining effective writing development is effective teaching (Graham, 2019), researchers have aimed to understand what writing instruction looks like across educational contexts, with several national surveys used to assess teachers’ preparation to teach writing, instructional practices, and efficacy beliefs to teach writing in primary education. In the US, primary (Grades 1–3) and elementary (Grades 4–6) teachers reported not being well prepared to teach writing in their pre-service training (Cutler & Graham, 2008; Gilbert & Graham, 2010). Findings indicated that teachers were using evidence-based practices to teach writing, but that these were applied infrequently, with teachers reporting allocating a small percentage of the school day for writing and emphasising the teaching of foundational writing skills, such as spelling and grammar usage, over teaching process skills associated with writing, such as planning and revising. In one of the few studies examining school and home connections, Cutler and Graham (2008) found that extended writing to the home environment was one of the least common practices for writing instruction. The vast majority of findings reported in the US have been replicated in more recent studies from different educational contexts across the globe.

Several national surveys developed in European countries have been reporting concerns about the teaching of writing in Grades 1–6. In a study examining the nature and frequency of writing instruction in England (Dockrell et al., 2016), teachers (Grades K-2) reported feeling confident in teaching writing and feeling prepared to teach it. However, nearly half of participating teachers reported difficulties in supporting struggling writers and spending more time teaching foundational skills, including spelling, than process skills, including revision strategies. In another study examining writing instruction in Portugal and in Brazil, findings indicated that Portuguese middle school teachers (Grades 5–9) also felt prepared to teach writing (Veiga-Simao et al., 2016). However, they reported allocating little time to writing instruction in their classrooms, infrequently using explicit teaching methods to teach writing and rarely focusing on the teaching of process skills, such as goal setting and revising. Results further indicated that teachers’ perceived preparation to teach writing correlated positively with their instructional practices; for example, teachers who felt better prepared to teach writing were more likely to report placing more attention to teaching students how to plan and revise their texts.

Findings from Dutch primary education contexts added knowledge to our understanding of writing instruction across the globe, with reports showing that, once again, the frequency of writing instruction was limited (Rietdik et al., 2018). Similar to findings from the US, 45% of the participating teachers (Grades 4–6) did not feel efficacious in teaching writing, and revision strategies were hardly included in their instructional practices. In a recent national survey examining writing instruction in Norway’s primary grades (Grades 1–3) (Graham, Skar, et al., 2021; Graham, Tavsanli, et al., 2021), findings once again replicated the low frequency of writing instruction and the lack of attention given to the teaching of process writing skills. Participant teachers also reported not feeling positive about their pre-service preparation but slightly to moderately positive about their efficacy to teach writing, with teachers’ preparation and efficacy values predicting reported instructional practices for writing.

Recent national surveys from South America and Asia have led to further insights on writing instruction across the globe, reinforcing concerns related to the nature and frequency of writing instruction in primary education (Banales et al., 2020; Hsiang & Graham, 2016; Hsiang et al., 2020). Findings from a national survey examining teachers’ preparation, instructional practices, and efficacy to teach writing in Chile (Grades 4–6) (Banales et al., 2020) indicated that the majority of teachers felt prepared to teach writing and that they were efficacious teachers of writing. Replicating reports from North American and European contexts, teachers reported spending less than five hours per week teaching writing, with results showing that teachers’ preparation and efficacy for teaching writing predicted their instructional practices, including time devoted to writing instruction and scaffolding of the writing process by teaching planning and revision strategies. Similar findings have been reported in studies examining writing instruction in the Great China region (Hsiang & Graham, 2016; Hsiang et al., 2020). In a comprehensive survey examining primary teachers (Grades 1–3) in Taiwan (Hsiang et al., 2020), a context with one of the highest rates of literacy scores on international comparative tests (OECD, 2016), the majority of teachers reported teaching writing just once a week or less. While teachers were not positive about their preparation to teach writing, they were more positive about their efficacy to teach it. Findings further showed that teachers’ beliefs accounted for 10–17% of the variance in reported instructional practices.

While the global picture of writing instruction in primary education highlights common concerns related to teachers’ preparation, instructional practices, and efficacy for teaching writing as reviewed here, much less is known about what writing instruction looks like in Oceanian countries. In a study examining writing instruction in New Zeeland (Parr & Jesson, 2016), findings suggested concerns related to the frequency of writing instruction in the primary grades, with Grade 1–8 teachers reporting spending approximately five hours per week teaching writing, but variability was relatively high. An effect of year level was also reported, with time teaching writing significantly lower for Grades 1–2. While the majority of teachers reported not being well prepared to teach writing from their pre-service training, teachers were overall confident in teaching writing, but less confident of their knowledge of out-of-school writing practices, including writing at home.

Empirical studies examining writing instruction in Australian primary classrooms are still scarce and state focused. In a comprehensive survey examining the teaching of writing in primary (Grades 1–6) and secondary classrooms (Grades 7–12) in New South Wales, Wyatt-Smith and colleagues (2018) found that primary teachers would invest more effort into teaching writing than secondary school teachers (33.1% versus 8.2%), with findings suggesting that across all the schooling stages the majority of teachers (over 50%) were more likely to not feel prepared for teaching writing. Malpique and colleagues (2017, 2020) examine the writing instruction provided in early primary classrooms in Western Australia. Their findings suggested a large variability in the teaching of foundational and process writing skills across kindergarten (Malpique et al., 2017) and Grade 1 classrooms (Malpique et al., 2020), with kindergarten and Grade 1 teachers reporting spending more time teaching foundational skills than process writing skills, with an emphasis on teaching spelling. The current exploratory study was developed to gain insights into the nature and frequency of writing instruction in Australia. This is, to our knowledge, the first national survey examining the teaching of writing in primary education in Australia.

### The current study

The current study investigated teachers’ reported instructional practices for writing in Grades 1–6, the compulsory grades of primary education in Australia. This included teachers’ preparation to teach writing; time allocated for writing practices and instruction; teachers’ perceived self-efficacy for teaching writing; specific practices supporting the development of students’ writing, including teaching strategies to extend writing to the home environment. We restricted assessment to specific evidence-based practices considered to play an important role in students’ writing development (Graham et al., 2015), identified as facilitating writing development in several meta-analyses examining the efficacy of instructional practices for writing in primary grades (e.g., Graham Harris & Santangelo, 2015; Graham et al., 2012; Graham et al., 2012). Teachers were surveyed between May 24, 2020, and November 1, 2020, during the first wave of the COVID-19 pandemic. During this period, Australia recorded cumulative confirmed cases of COVID-19, with each state experiencing different degrees of severity, subsequent lockdowns, and education interruptions. To mitigate the potential effects of the pandemic on teachers’ reports, we asked them to report on *typical* (in school) practices they followed for teaching writing when completing the survey. Of note is the fact that education interruptions were dependent on the degree of severity of the pandemic, with school closures affecting Australian states and territories differently (Leask & Hooker, 2020).

Our work extends research in the field in two important ways. First, previous surveys have allocated little attention to examining teaching strategies to extend writing to the home environment. In one of the few studies examining this, Cutler and Graham (2008) found that primary teachers in the US did not make strong connections for writing between home and school. Considering the inconsistent educational structures and practices developed for remote learning during the first wave of COVID-19 and the role that families were asked to play to support learning (Fitzgerald et al., 2020), it becomes important to understand how frequently teachers typically develop school/home relationship designed to foster students’ writing development (Merga et al., 2021). Second, research indicates that teachers’ preparation and self-efficacy for teaching writing are the two most consistent predictors of teachers’ writing practices (Banales et al., 2020; Graham, 2019). However, little is known about the unique role that these two teacher variables play in predicting the development of school/home connections to promote effective writing.

The current study addressed the following research questions:

1. How much time do teachers allocate for writing practices and writing instruction in Australian primary classrooms (Grades 1–6)?
2. Do primary teachers find they are prepared to teach writing?
3. Do primary teachers find they are effective at teaching writing?
4. What instructional practices do primary teachers use to support students’ writing?
5. Do teachers’ perceived efficacy at teaching writing and their preparation to teach writing make a unique and significant contribution to predicting the reported instructional practices for writing?

Based on previous research examining teachers’ reported instructional practices for writing in Grades 1–6, we anticipated that teachers would report allocating less than the recommended 30 min per day to writing practice in Australian primary classrooms (Graham, Bollinger, et al., 2012; Graham, McKeown, et al., 2012). We further expected that teachers would report applying a multitude of evidence-based instructional practices for writing to promote writing development. Based on previous research developed across different educational contexts, however, we anticipated that these instructional practices would not be frequently implemented, and that a stronger focus would be placed on teaching foundational skills associated with writing (e.g., US, Cutler & Graham, 2008; Dockrell et al., 2016; Graham et al., 2021; Graham, Tavsanli et al., 2021).

In the current study, we examined teachers’ preparation for teaching writing. Multiple studies have shown that increasing teachers’ preparation to teach writing impacted students’ writing performance positively (e.g., Gallagher et al., 2017; Wolbers et al., 2017). We also examined teachers’ perceived self-efficacy for teaching writing. Teachers’ confidence to design and implement activities that promote learning was found to predict teachers’ practices and students’ outcomes (Holzberger et al., 2013; Yada et al., 2019). Multiple studies have also found relationships between teachers’ self-efficacy and reported instructional practices for writing (e.g., Banales et al., 2020; Veiga Simao et al., 2016). Taken together, these studies substantiated the focus placed on teachers’ preparation and self-efficacy for teaching writing in this study.

Our predictions regarding teachers’ preparation and efficacy for teaching writing were not, however, straightforward. Previous research examining teachers’ preparation describe different outcomes, with primary teachers in the US, Norway, the Netherlands, Taiwan, and New Zeeland reporting not being well prepared to teach writing, as opposed to English, Portuguese, and Chilean teachers. Similar opposing findings across educational contexts have been described regarding teachers’ perceived efficacy for teaching writing in primary education, with teachers from England, Chile, Taiwan, Portugal, and New Zeeland reporting feeling overall confident in teaching writing (i.e., Dockrell et al., 2016; Banales et al., 2020; Hsiang et al., 2020; Veiga-Simao et al., 2016; Parr & Jesson, 2016), as opposed to teachers in the US and in the Netherlands, for example (i.e., Cutler & Graham, 2008; Rietdik et al., 2018). Based on previous studies (Banales et al., 2020; Hsiang et al., 2020; Veiga Simao et al., 2016), we expected that both teachers’ reported preparation and perceived efficacy for teaching writing would be positively related to teachers’ instructional practices.

Teacher and classroom variables have been found to account for variability in teachers’ reported practices for writing (e.g., Banales et al., 2020; Graham, 2019). Hence, we controlled for teacher (i.e., gender; years spent teaching; and educational level) and class (i.e., grade level taught) variables to examine variance in the reported instructional practices for writing. We controlled for teachers’ gender since several studies found relationships between teachers’ gender, teachers’ practices and students’ outcomes (see Sabbe & Aelterman, 2007, for a review); we controlled for teaching experience (reported number of years teaching in primary classrooms), found to statistically account for variability in teachers’ reported writing practices (Banales et al., 2020); we further controlled for teachers’ educational level since teachers’ pedagogical knowledge is commonly enhanced via academic and professional development (Veiga Simao et al., 2016), and teachers’ knowledge and experience was found to influence classroom writing practices (Graham & Harris, 2017); finally, we controlled for grade level considering findings suggesting grade level effects on teachers’ reported practices for writing (Parr & Jesson, 2016).

## Method

### Participants and setting

Primary school teachers (Grades 1–6) across states and territories in Australia were invited to complete an online survey designed to assess writing instruction in primary education. The first phase of the recruitment process consisted of contacting all principals of the 1449 non-governmental public schools compiled by the Australian Curriculum, Assessment and Reporting Authority (ACARA, nd). Principals were asked to share the information letter containing the online survey with all primary teachers in their schools. We further contacted relevant professional associations (e.g., Australian Primary Principals Association and Primary English Teaching Association Australia) to advertise the project and recruit further participants.

The current survey had N = 310 respondents, and we used GPower (Faul et al., 2009) to determine a minimum sample size needed for statistical analysis. We then applied Cohen’s convention of a medium effect size threshold of 0.30 (Cohen, 2013), with a 95% confidence interval, and found that the suggested threshold would be N = 196, a figure comfortably exceeded by the N = 310 sample in this instance. The sample did not include special education teachers. The online survey was open to all primary teachers from Grades 1 to 6 since all teachers in Australia are expected to teach writing skills across grades (ACARA, 2021).

Writing instruction in Australia is guided by achievement benchmarks for writing development, which are set at a national level, with states and territories responsible for implementing an adapted version of the national curriculum (ACARA, 2021). In essence, achievement standards are set under the English subject strand of the Australian national curriculum, with different versions operating across states and territories (Weatherby-Fell, 2015). Standards are set for the development of foundational writing skills (e.g., handwriting, typing, and spelling) and process writing skills (e.g., planning, organising, and editing texts). Moreover, under the Australian curriculum, literacy is presented as a general capability (ACARA, nd), and all teachers across primary and secondary grades are expected to support the development of literacy skills, including writing. However, specific instructional practices for writing are not mandated by national and state entities, highlighting the importance of gaining insights about teaching practices currently being implemented to support writing development in Australian classrooms.

### Instrumentation

Teachers were asked to respond to items on an online survey constructed to examine writing instruction in primary education in Australia. To generate items for the online survey, reviews were performed to examine previous surveys assessing writing instruction in Grades 1–6 conducted in other educational contexts. The current survey was adapted from two national surveys on writing instruction developed in the US (Cuttler & Graham, 2008; Gilbert & Graham, 2010). In developing the survey, we chose specific scales and condensed some scales to answer the proposed research questions. The survey was field tested prior to its use in this study. Four primary grade teachers completed the survey and were asked to provide feedback on an initial version. We conducted open-ended interviews with the respondents to identify difficulties arising in its administration (e.g., language issues, questions that could hinder participants’ responses, and time to complete it) and to evaluate their interpretation of the items. We made several changes based on subsequent analyses, namely on item clarity and wording.

The survey applied in this investigation included 42 items grouped into five main sections: (1) teacher information (8 items); (2) time for writing practice and teaching writing (7 items); (3) teachers’ self-efficacy for teaching writing (5 items); (4) teachers instructional practices supporting the development of students’ writing (20 items); (5) impact of COVID-19 on teachers’ typical teaching practices (2 items). In the first section, we asked teachers to provide demographic information including their gender, highest educational level and years spent teaching. Teachers were also asked to rate the quality of their pre-service preparation to teach writing. In the second section, teachers were prompted to think about their typical practices for teaching writing and to indicate how much time they allocated on average to writing practice in their classrooms; they were also asked to indicate how much time they allocated to teaching foundational skills (i.e., handwriting, typing, spelling, and grammar usage) and process skills (i.e., revision strategies and planning strategies).

The third section assessed teachers’ self-efficacy for teaching writing using a 5-item scale adjusted from the 9-item scale applied by Gilbert and Graham (2010). Teachers reported on the perceived efficacy to teach writing and to adjust teaching practices to students’ specific needs. Teachers responded to this section using a six-point Likert-type scale ranging from 1 (*Strongly Disagree*) to 6 (*Strongly Agree*), with high scores indicating a stronger sense of efficacy to teach writing. A factor analysis produced a single factor with an eigenvalue greater than 1.0 explaining 67% of the overall variance, with all factors loaded at 0.76 or greater. The coefficient alpha value for this factor was 0.83. The score for self-efficacy for teaching writing was the average of the five items.

The fourth section examined teachers’ instructional practices supporting the development of students’ writing. The 20 items in this section were adapted from Cutler and Graham’s survey (2008) examining writing instruction in the US. The items focused on four aspects of writing instruction, namely evidence-based practices to promote effective writing; teaching strategies supporting the development of foundational writing skills; teaching strategies supporting the development of process writing; and teaching practices to extend writing to the home environment. Teachers were asked to indicate how often they typically included specific practices for writing in their classrooms and responded to this section using a five-point Likert-type scale ranging from 1 (*Never*) to 5 (*Daily*). A factor analysis produced four factors with an eigenvalue greater than 1.0 explaining 58% of the overall variance. A Varimax rotation was also performed to maximise factor dispersion and to produce a more interpretable solution. Six items loaded at 0.69 or greater on the first factor, identified as evidence-based practices to promote effective writing, with a coefficient alpha value of 0.85; six items loaded at 0.65 or greater on the second factor, identified as teaching strategies supporting the development of foundational skills, with a coefficient alpha value of 0.84; four items loaded at 0.74 or greater on the third factor, identified as teaching strategies supporting the development of process writing, with a coefficient alpha value of 0.93; and four items loaded at 0.69 or greater on the fourth factor, identified as teaching practices to extend writing to the home environment, with a coefficient alpha value of 0.70. The score for each factor was the average of the included items.

The fifth section of the survey asked teachers to indicate if COVID-19 had affected their typical teaching practices for writing. In the first item, teachers were asked to indicate if the pandemic had affected writing instruction in their current class. Skip logics were programmed into the survey that only respondents who indicated that COVID-19 was affecting writing instruction were directed to a second open-ended and explanatory question, in which teachers were asked to provide further information on how COVID-19 was affecting their typical writing practices. A final section of the survey asked teachers to provide additional information about their writing program via open-ended response.

## Results

Respondents were from a range of geographic contexts within Australia, as per Table 1 below. The majority of participating teachers were female (87%) and held a bachelor’s degree or a graduate diploma (81%). Though all states and territories were represented in the survey responses, the majority of respondents (61%) taught in Australian states where there were fewer education interruptions during the first wave of COVID-19 (i.e., Northern Territory, Western Australia, South Australia, and Tasmania). The representation of responses across primary grades was relatively even.

**Table 1:** Respondent characteristics

Variable	In sample (*n* = 310)	In sample (%)
*Gender of teacher*		
Female	272	87.7
Male	38	12.3
Other	–	–
*State/Territory where teaching*		
ACT	27	8.7
NSW	48	15.5
NT	22	7.1
QLD	31	10
SA	50	16.1
TAS	35	11.3
VIC	15	4.8
WA	82	26.5
*Highest degree achieved*		
Vocational	5	1.6
Bachelor	179	57.7
Graduate Diploma	78	25.2
Masters	44	14.2
Doctorate	4	1.3
Years of teaching		
Mean	15.15	–
Median	15	–
*Grade(s) currently taught*		
1	42	13.5
2	56	18.1
3	47	15.2
4	60	19.4
5	57	18.4
6	48	15.5

### Time for teaching writing and writing practice

Teachers reported allocating some time for writing practice in their classrooms and to teach foundational and process writing skills on a weekly basis. On average, teachers reported allocating nearly three hours a week for writing practice in their classrooms (*M* = 168.77 min, *SD* = 91.26; range = 15–450 min). They further reported teaching foundational skills, allocating more time a week teaching spelling (*M* = 88.13 min, *SD* = 47.21; range = 15–400 min) than the remaining foundational skills, namely teaching handwriting (*M* = 34.35 min, *SD* = 26.44; range = 0–240 min; *t*(309) = 22.75, *p* = 0.000,* d* = 1.40); teaching grammar (*M* = 54.66 min, *SD* = 37.34; range = 5–400 min; *t*(309) = 15.00, *p* = 0.000,* d* = 0.79); and teaching typing (M = 11.24 min, SD = 21.05; range = 0–300 min; *t*(309) = 27.00, *p* = 0.000,* d* = 2.10). Teachers also reported allocating more time a week teaching grammar than teaching handwriting (*t*(309) = 10.87, *p* = 0.000,* d* = 0.63); and typing (*t*(309) = 19.41, *p* = 0.000,* d* = 1.43). Time devoted to teaching process writing skills was less than for teaching foundational skills, expect for teaching typing. On average, teachers reported allocating more time teaching spelling than teaching planning (*M* = 35.48 min, *SD* = 25.54; range = 0–180 min; *t*(309) = 21.86, *p* = 0.000,* d* = 1.39) and revision strategies (*M* = 42.71 min, *SD* = 28.53; range = 0–200 min; *t*(309) = 16.84, *p* = 0.000,* d* = 1.16); more time teaching grammar than teaching planning (*t*(309) = 11.89, *p* = 0.000,* d* = 1 = 0.60) and revision strategies (*t*(309) = 19.41, *p* = 0.000,* d* = 0.34); and more time teaching handwriting than revison strategies (*t*(309) = 4.74, *p* = 0.000,* d* = 0.15). Finally, time devoted to teaching process writing skills was less for teaching planning than revision strategies (*t*(309) = 4.74, *p* = 0.000,* d* = 0.27).

### Teachers’ reported preparation and self- efficacy for teaching writing

Teachers indicated they were generally positive about their preparation to teach writing. A higher percentage of teachers reported they had received adequate pre-service training to teach writing (41.70%), with another 27.69% reporting their pre-service preparation was very good. About 30% of teachers, however, believed their undergraduate preparation was poor (27.36%) or inadequate (2.28%). Teachers reported feeling moderately confident about their efficacy to teach writing (*M* = 4.90, *SD* = 0.64), and efficacy scores indicated that 27, 52% and 21% were strongly, moderately, and slightly confident about their capabilities to teach writing. Forty-seven percent of participating teachers reported feeling slightly confident or less about their efficacy to adjust teaching practices to students’ specific needs.

### Evidence-based practices to promote effective writing

Frequencies, means and standard deviations for the six evidence-based practices to promote effective writing are presented in Table 2. The majority of participating teachers reported their students engaged in planning and revising their writing at least once a week (i.e., 51 and 57.4%, respectively), but only sharing their writing with their peers once a month or less (58.4%). The majority of teachers reported overtly modelling writing strategies (64.8%), modelling enjoyment for writing (80.3%), and sharing their own writing with their students (92.3%) once a month or less. Of note is that less than 9% of the participating teachers reported including the assessed six evidence-based practices to support effective writing on a daily basis.

**Table 2:** Writing practices and instructional procedures

	Never (%)	At least once a year (%)	At least once a month (%)	At least once a week (%)	Daily (%)	*M* (*SD*)
*Evidence-based practices*						
Students engage in planning before writing	1.6	10.0	37.4	42.9	8.1	3.46 (0.84)
Students revise their writing products	1.3	6.1	35.2	50.0	7.4	3.56 (0.77)
Students share their writing with their peers	1.0	13.5	43.9	38.1	3.5	3.30 (0.78)
Teacher sharing own writing	52.9	23.9	15.5	4.2	3.5	1.82 (1.07)
Teacher overtly modelling writing strategies	4.5	23.2	37.1	26.8	8.4	3.11 (1.00)
Teacher modelling enjoyment for writing	26.1	25.8	28.4	13.5	6.1	2.48 (1.19)
*Foundational writing skills*						
Teaching spelling	0.0	1.0	19.0	44.2	35.8	4.15 (0.75)
Teaching grammar	0.0	5.5	33.2	51.9	9.4	3.65 (0.72)
Teaching punctuation	0.0	8.4	37.7	45.5	8.4	3.54 (0.76)
Teaching capitalisation	0.6	6.8	32.3	48.4	11.9	2.91 (1.03)
Teaching handwriting	9.0	27.4	30.0	30.3	3.2	1.80 (1.07)
Teaching typing	54.5	24.5	8.4	11.6	1.0	3.63 (0.80)
*Process writing skills*						
Teaching sentence skills	4.5	25.2	35.2	30.3	4.8	3.06 (0.96)
Teaching how to organise texts	3.2	27.1	32.6	36.8	0.3	3.04 (0.88)
Teaching strategies for planning	10.6	35.2	33.5	20.6	0.0	2.64 (0.93)
Teaching strategies for revising and editing	5.2	36.5	40.6	16.5	1.3	2.72 (0.84)
*School-home connections*						
Assigning writing homework	37.7	26.1	28.4	7.7	0.0	2.06 (0.98)
Asking students to write at home with the assistance of a parent/ guardian	64.8	22.9	8.4	3.2	0.6	1.52 (0.83)
Asking parents/guardians to read something their child wrote at school	24.8	52.6	18.7	3.9	0.0	2.02 (0.77)
Communicating (formally or informally) with parents/guardians	1.0	71.0	25.2	2.9	0.0	2.30 (0.77)

### Teaching strategies supporting the development of foundational and process writing skills

Regarding the frequency with which teachers taught foundational writing skills, the majority of teachers (80%) reported teaching spelling once a week or more often. Grammar, punctuation, and capitalisation skills were also reportedly taught once a month or more often by a majority of teachers (i.e., 60, 54%, and 60%, respectively). Teachers reported spending less time teaching handwriting, with only 34% of teachers indicating that they taught handwriting skills once a week or more often, and 9% of teachers reporting never including the teaching of handwriting in their instructional practices for writing. The majority of teachers (54.5%) reported that they never taught typing skills.

Results assessing the frequency of teaching practices supporting the development of process writing skills indicated that the majority of teachers reported they taught sentence skills and organisational features of texts only once a month or less (i.e., 61 and 60%, respectively). Teaching strategies for planning and revising were the least included in teachers’ reported instructional practices, with the majority of teachers (77%) indicating that they taught strategies for revising and planning once a month or less (i.e., 77 and 69%, respectively). Of note is that 11% of teachers reported never teaching planning strategies for writing.

### Teaching practices to extend writing to the home environment

Results assessing reported teaching practices to extend writing to the home environment showed that the majority of teachers (64.8%) never asked students to write at home with the support of a parent/guardian. The majority of teachers (77.4%) also reported that they asked parents/guardians to read their children’s written work once a year or never. The majority of teachers (63.9%) reported never or only yearly assigning writing homework. Communicating with parents/guardians to discuss students’ performance and needs was reported as an infrequent practice by the majority of participating teachers (71.9%). The majority of teachers (72.6%) reported that COVID-19 had affected writing instruction in their class(es).

### Grade level variability

For each of the 20 writing practices and instructional procedures assessed in this study, a separate one-way ANOVA was conducted to determine whether teachers across the six primary grades (1–6) differ in how often they applied the practices. To control for Type 1 errors, we made a Bonferroni adjustment (α of 0.05/20 analyses), setting alpha at 0.002. Grade-level was not statistically related to mean scores for 15 of the assessed writing practices and instructional procedures (all *p*’s > 0. 003). Grade-level was statically related to mean scores for students engaged in planning before writing (*F*(5,304) = 6.440, *p* = 000); students revising their writing products (*F*(5,304) = 7.513, *p* = 000); teaching spelling (*F*(5,304) = 13.253, *p* = 000; teaching capitalisation (*F*(5,304) = 4.242, *p* = 001); and teaching handwriting (*F*(5,304) = 18.442, *p* = 000. For students engaged in planning before writing, first grade (*M* = 2.93; *SD* = 1.28) had statically lower scores than fourth (*M* = 3.57; *SD* = 0.79), fifth (*M* = 3.63; *SD* = 0.62), and sixth grades (*M* = 3.75; *SD* = 0.76). For students revising their writing products, first grade (*M* = 2.93; *SD* = 1.06) had statically lower scores than fifth (*M* = 3.81; *SD* = 0.48), and sixth grades (*M* = 3.81; *SD* = 0.67); second grade (*M* = 3.27; *SD* = 0.84) also had statically lower scores than fifth grade. For teaching spelling, sixth grade (*M* = 3.56; *SD* = 0.68) had statically lower scores than first (*M* = 4.55; *SD* = 0.63); second (*M* = 4.50; *SD* = 0.69); third (*M* = 4.15; *SD* = 0.59); and fourth grades (*M* = 4.00; *SD* = 0.73). For teaching capitalisation, sixth grade (*M* = 3.29; *SD* = 0.90) had statically lower scores than first grade (*M* = 4.00; *SD* = 0.70). For teaching handwriting, sixth grade (*M* = 2.08; *SD* = 0.92) had statically lower scores than first (*M* = 3.50; *SD* = 0.77); second (*M* = 3.32; *SD* = 0.92); third (*M* = 3.19; *SD* = 0.85); and fourth grades (*M* = 3.05; *SD* = 1.10).

### Predicting the reported classroom practices for writing

To determine whether teachers’ perceived efficacy at teaching writing and their preparation to teach writing made a unique and significant contribution to predicting the reported classroom practices for writing (evidence-based practices, foundational skills, process skills, and school-home connections), beyond that already accounted for by teacher and class variables (gender, years spent teaching, highest educational level, grade currently taught and COVID-19 impact), four hierarchical multiple regression analyses (MRA) were employed. In step 1 of the hierarchical MRA, teacher and class variables were added. In step 2, teachers’ self-efficacy and preparation were added to the regression equation. Unstandardised (*B*) and standardised (β) regression coefficients for each predictor on each step of the hierarchical MR are reported in Tables 3, 4, 5 and 6.

**Table 3:** Unstandardised (B) and standardised (β) regression coefficients and squared semi-partial correlations (sr^2^) for each predictor variable on each step of a hierarchical multiple regression predicting evidence-based practices

Variable	*B* (95% CI)	β	*sr*^*2*^
*Step 1*			
Gender	0.27 [ 0.02, 0.52]*	0.12	0.12
Years of teaching	0.00 [− 0.01, 0.01]	0.05	0.05
Highest educational level	0.01 [− 0.10, 0.11]	0.01	0.01
Grade currently taught	0.04 [− 0.01, 0.10]	0.10	0.10
COVID-19	0.05 [− 0.13, 0.23]	0.03	0.03
*Step 2*			
Gender	0.13 [− 0.10, 0.35]	0.06	0.06
Years of teaching	− 7.60 [− 0.01, 0.01]	0.00	0.00
Highest educational level	− 0.05 [− 0.14, 0.05]	− 0.05	− 0.06
Grade currently taught	0.02 [− 0.03, 0.07]	0.05	0.05
COVID-19	0.04 [− 0.12, − 0.21]	0.03	0.03
Self-efficacy	0.59 [ 0.45, 0.72]***	0.52	0.46
Preparation	0.16 [ 0.06, 0.26]**	0.19	0.18

*CI* Confidence interval

*p < 0.05. **p < 0.01. ***p < 0.001

**Table 4:** Unstandardised (B) and standardised (β) regression coefficients and squared semi-partial correlations (sr^2^) for each predictor variable on each step of a hierarchical multiple regression predicting foundational skills

Variable	*B* (95% CI)	β	*sr*^*2*^
*Step 1*			
Gender	0.12 [− 0.10, 0.34]	0.03	0.03
Years of Teaching	0.00 [− 0.01, 0.01]	0.05	0.05
Highest Educational Level	0.06 [− 0.04, 0.15]	0.07	0.07
Grade Currently Taught	− 0.13 [− 0.18, -0.09]***	− 0.34	− 0.32
COVID-19	0.20 [ 0.04, 0.36]*	0.14	0.03
*Step 2*			
Gender	0.06 [− 0.16, 0.27]	0.06	0.06
Years of Teaching	0.01 [− 0.00, 0.01]	0.07	0.07
Highest Educational Level	0.04 [− 0.05, 0.13]	0.05	0.05
Grade Currently Taught	−0.14 [− 0.18, -0.10]***	− 0.35	− 0.34
COVID-19	0.15 [− 0.01, 0.20]	0.10	0.11
Self-Efficacy	0.21 [ 0.09, 0.34]**	0.20	0.19
Preparation	0.19 [ 0.09, 0.29]***	0.24	0.22

*CI* Confidence interval

*p < 0.05. **p < 0.01. ***p < 0.001

**Table 5:** Unstandardised (B) and standardised (β) regression coefficients and squared semi-partial correlations (sr^2^) for each predictor variable on each step of a hierarchical multiple regression predicting process skills

Variable	*B* (95% CI)	β	*sr*^*2*^
*Step 1*			
Gender	0.16 [ − 0.12, 0.45]	0.06	0.07
Years of teaching	0.01 [ − 0.00, 0.02]	0.11	0.11
Highest educational level	0.04 [ − 0.08, 0.16]	0.04	0.04
Grade currently taught	0.03 [ − 0.03, 0.09]	0.07	0.06
COVID-19	0.12 [ − 0.09, 0.32]	0.06	.07
*Step 2*			
Gender	0.02 [ − 0.25, 0.30]	0.01	0.01
Years of teaching	0.01 [ − 0.00, 0.02]	0.00	0.00
Highest educational level	− 0.01 [ − 0.12, 0.11]	0.09	0.09
Grade currently taught	0.02 [ − 0.04, 0.07]	0.03	0.03
COVID-19	0.07 [ − 0.13, 0.27]	0.04	0.04
Self-Efficacy	0.51 [ 0.35, 0.67]***	0.39	0.34
Preparation	0.24 [ 0.12, 0.36]***	0.24	0.21

*CI* Confidence interval

*p < 0.05. **p < 0.01. ***p < 0.001

**Table 6:** Unstandardised (B) and standardised (β) regression coefficients and squared semi-partial correlations (sr^2^) for each predictor variable on each step of a hierarchical multiple regression predicting school-home connections

Variable	*B* (95% CI)	β	*sr*^*2*^
*Step 1*			
Gender	0.08 [− 0.10, 0.27]	0.05	0.05
Years Of teaching	0.01 [ 0.00, 0.02]	0.13	0.13
Highest educational level	0.00 [− 0.08, 0.08]	0.00	0.00
Grade currently taught	− 0.01 [− 0.05, 0.03]	− 0.02	− 0.02
COVID-19	− 0.04 [− 0.17, 0.10]	− 0.03	− 0.03
*Step 2*			
Gender	0.02 [− 0.16, 0.19]	0.01	0.01
Years of teaching	0.00 [− 0.00, 0.01]	0.06	0.06
Highest educational level	− 0.03 [− 0.10, 0.04]	− 0.04	− 0.05
Grade currently taught	− 0.02 [− 0.06, 0.02]	− 0.07	− 0.07
COVID-19	− 0.00 [− 0.13, 0.13]	− 0.00	− 0.00
Self-Efficacy	0.30 [ 0.20, 0.41]***	0.37	0.32
Preparation	− .012 [− 0.09, 0.06]	− 0.02	− 0.02

*CI* Confidence interval

*p < .05. **p < 0.01. ***p < 0.001

First, a missing value analysis was conducted, with three participants having missing data. Little’s (1998) MCAR test was not significant (χ^2^ (5) = 9.48, *p* > 0.05), indicating that the data were missing completing at random. Therefore, the full information maximum likelihood technique was used to estimate the data (Enders, 2010).

Before interpreting the results of the MRA, assumptions were tested, and checks were performed. First, stem-and-leaf plots and boxplots indicated that each variable in the regression was normally distributed. However, a total of six outliers were identified and therefore deleted (Tabachnick & Fidell, 2013). Second, an inspection of the normal probability plot of standardised residuals and the scatterplot of standardised residuals against standardised predicted values indicated that the assumptions of normality, linearity and homoscedasticity of residuals were met. Third, the Mahalanobis distance exceeded the critical χ^2^ for *df* = 7 (at α = 0.001) of 27.87 for only one case in the data file, indicating the presence of one multivariate outlier. Following the decision to delete the associated case (Tabachnick & Fidell, 2013), multivariate outliers were no longer of concern (χ^2^ (7) = 21.61, *p* < 0.001). Finally, relatively high tolerances for all predictors in the final regression model indicated that multicollinearity was not of concern.

#### Evidence-based practices to promote effective writing

In step one of the hierarchical MRA, teacher and class variables (gender, years spent teaching, highest educational level, grade currently taught, and COVID-19) accounted for a non-significant 3.3% of the variance in teachers’ use of evidence-based practices, *R*^2^ = 0.03, *F* (5, 297) = 2.03, *p* = 0.074. In step two, teachers’ self-efficacy and preparation were added to the regression equation and accounted for an additional 20.1% of the variance in teachers’ use of evidence-based practices, $\Delta$ *R*^2^ = 0.20, $\Delta$ *F* (2, 295) = 38.66, *p* < 0.001. In combination, the predictor variables explained 23.4% of the variance in teachers use of evidence-based practices, *R*^2^ = 0.23, adjusted *R*^2^ = 0.22, *F* (7, 295) = 12.86, *p* < 0.001. However, only teachers’ self-efficacy and preparation made a unique and statistically significant contribution to predicting teachers’ reported use of evidence-based practices (Table 3). Hence, teachers who believed they were more efficacious and prepared were more likely to report that they included the assessed evidence-based practices to support effective writing in their classrooms.

#### Teaching foundational skills

In step one of the hierarchical MRA, teacher and class variables (gender, years spent teaching, highest educational level, grade currently taught and COVID-19) accounted for a significant 13.2% of the variance in teaching foundational skills, *R*^2^ = 0.13, *F* (5, 297) = 9.03, *p* < 0.001. In step two, teachers’ self-efficacy and preparation were added to the regression equation and accounted for an additional 5.2% of the variance in teaching foundational skills, $\Delta$ *R*^2^ = 0.05, $\Delta$ *F* (2, 295) = 9.35, *p* < 0.001. In combination, the predictor variables explained 18.4% of the variance in teaching foundational skills, *R*^2^ = 0.18, adjusted *R*^2^ = 0.16, *F* (7, 295) = 9.48, *p* < 0.001. However, only grade currently taught, teachers’ self-efficacy and teachers’ preparation made a unique and statistically significant contribution to predicting the reported teaching of writing foundational skills (Table 4). Thus, teachers who taught lower grade levels and teachers who believed they were more efficacious and better prepared were more likely to report that they taught strategies supporting the development of foundational skills.

#### Teaching process skills

In step one of the hierarchical MRA, teacher and class variables (gender, years spent teaching, highest educational level, grade currently taught and COVID-19) accounted for a non-significant 3.2% of the variance in teaching process skills, *R*^2^ = 0.03, *F* (5, 297) = 1.99, *p* = 0.080. In step two, teachers’ self-efficacy and preparation were added to the regression equation and accounted for an additional 12% of the variance in teaching process skills, $\Delta$ *R*^2^ = 0.12, $\Delta$ *F* (2, 295) = 20.85, *p* < 0.001. In combination, the predictor variables explained 15.2% of the variance in teaching process skills, *R*^2^ = 0.15, adjusted *R*^2^ = 0.13, *F* (7, 295) = 20.85, *p* < 0.001. However, only teachers’ self-efficacy and preparation made a unique and statistically significant contribution to predicting teaching process skills (Table 5). In other words, teachers who believed they were more efficacious and better prepared were more likely to report that they taught strategies supporting the development of process skills.

#### Developing school-home connections

In step one of the hierarchical MRA, teacher and class variables (gender, years spent teaching, highest educational level, grade currently taught and COVID-19) accounted for a non-significant 2.1% of the variance in school-home connections, *R*^2^ = 0.02, *F* (5, 297) = 1.30, *p* = 0.265. In step two, teachers’ self-efficacy and preparation were added to the regression equation and accounted for an additional 12.6% of the variance in school-home connections, $\Delta$ *R*^2^ = 0.13, $\Delta$ *F* (2, 295) = 21.73, *p* < 0.001. In combination, the predictor variables explained 14.7% of the variance in school-home connections, *R*^2^ = 0.15, adjusted *R*^2^ = 0.13, *F* (7, 295) = 7.27, *p* < 0.001. However, only teachers’ self-efficacy made a unique and statistically significant contribution to predicting school-home connections (Table 6). Hence, teachers who believed they were more efficacious were more likely to report that they developed practices to extend writing to the home environment.

## Discussion

### Time for writing and instructional practices

Effective writing development is a function of explicit teaching and practice (Applebee & Langer, 2006; Kellogg, 2008). Findings from the current study suggested that Australian primary students are spending about three hours on writing practice per week on average, the minimum time recommended for writing practice in primary education (Graham et al., 2012; Graham et al., 2012). Large variability was reported in the amount of time students spend writing, ranging from 15 min to 7.5 h per week. Similar findings were reported in the few studies examining writing instruction in Australian classrooms (Malpique et al., 2017, 2020). When investigating the writing instruction provided in kindergarten and Year 1 classrooms, Malpique and colleagues noticed that the teaching of writing varied rather noticeably in both years of schooling, ranging from 20 to 300 min in kindergarten and 60–360 min in Year 1.

Research on writing instruction has consistently reported a large variation in time for writing and writing practices in primary education, both in national surveys (e.g., Cuttler & Graham, 2008) and in observational studies (e.g., Coker Jr et al., 2016). Providing adequate writing practice is an essential cornerstone of writing instruction since it enables students to have more opportunities to develop the writing skills and confidence to face different writing tasks while, simultaneously, it gives teachers more opportunities to identify writing difficulties at the outset and develop strategies to respond to students’ needs (Graham et al., 2012; Graham et al., 2012). While this apparent lack of uniformity in writing instruction in Australian classrooms replicates previous national and international research, little is known about its impact on students’ writing development. Despite being consistent with the premise that there is variability across writing communities in which writing is developed, as proposed in the WWC model (Graham, 2018), research investigating relationships between variations in writing practices, teachers’ beliefs, knowledge, and values about writing is clearly needed.

Findings from our study further suggest that Australian primary teachers allocate more time to teaching foundational skills than process writing skills. In the second section of our study, we asked teachers to report on the time they usually spent teaching foundational and process skills in their classrooms. The participating teachers reported spending more time teaching spelling (85 min per week) and teaching grammar (55 min per week). By contrast, time reportedly devoted to teaching revision and planning strategies was less (42 and 35 min, respectively). In the fourth section of our study, we asked teachers to report on the use of practices supporting writing development in primary education. Of the 20 different practices included in the survey, the majority of teachers reported that they implemented six of them at least weekly and all of the remaining practices were reportedly applied less frequently or never. The majority of teachers reported teaching spelling (80%), grammar (62%), punctuation (54%) and capitalisation (60%) at least weekly. By contrast, the majority of teachers reported teaching sentence skills (60%), text organising skills (60%), planning strategies (69%), and revising strategies (77%) once a month or less. We also found grade level variability in five writing practices and instructional procedures examined. Findings indicated that more attention was allocated to the teaching of some foundational skills, namely spelling and handwriting, in the lower primary grades (1–3). Our findings further suggest that upper primary teachers (Grades 4–6) allocated more opportunities for students to plan and revise their writings than lower primary teachers.

The findings from our study are consistent with previous findings from the US (Cutler & Graham, 2008), England (Dockrell et al., 2016) and Norway (Graham et al., 2021; Graham et al., 2021), where primary school teachers (Grades 1–3) reported placing a stronger emphasis on teaching basic skills associated with writing. Cross-sectional studies investigating writing development and instruction in Australia also found a marked variation in the teaching of foundational skills and process writing skills in kindergarten (Malpique et al., 2017) and in Year 1 (Malpique et al., 2020), with results suggesting that teaching spelling was the type of writing instruction more frequently included in the first two years of formal schooling in Australia. Research findings indicate a positive impact of explicitly teaching process writing skills on primary students’ writing quality (average weighted effect size *d* = 1.02, 20 studies, grades 2–6; Graham et al., 2012; Graham et al., 2012). As such, our findings are worrisome when considering that evidence-based recommendations for teaching writing suggest that the teaching of foundational skills and process writing skills need to be included in tandem in weekly writing instruction across Grades 1 to 6 (Berninger et al., 1997; Graham et al., 2012a; Graham et al., 2012; Malpique & Veiga-Simao, 2019).

Of note is that our findings suggest that less focus is placed on teaching handwriting and typing in Australian primary classrooms. Handwriting instruction was reported to occur only 30 min per week, whereas typing instruction was a much rarer occurrence (11 min per week), with 54.5% of teachers reporting that they never taught typing in their classrooms. Similar findings have been reported in studies examining writing instruction in the US (Gilbert & Graham, 2010) and in England (Dockrell et al., 2016), for example. Reports on primary teachers’ preparation to teach writing in New South Wales indicate that both transcriptions skills were not emphasised in initial teacher education in this Australian state, with only 39% of primary teachers indicating that they had explicit instruction in teaching handwriting and 44% of primary teachers indicating that they were not prepared to teach typing (Wyatt-Smith et al., 2018).

Moreover, our findings indicate that lower primary teachers (Grades 1–3) place a stronger focus on teaching handwriting than upper primary teachers (Grades 4–5). These findings are well aligned with studies showing that explicit handwriting instruction takes place only in the first years of schooling, despite empirical evidence showcasing the validity of handwriting instruction beyond the first years (see Limpo & Graham, 2020, for a review). Substantiated by the capacity theory of writing (McCutchen, 1995), effective writing relies heavily on the writer’s ability to access and retrieve alphabet letters in memory and automatically. Lack of fluency in letter writing may constrain higher-level writing processes, including planning and revising (Jansen et al., 2017). Whether via paper and pencil or typing, proficient automatic writing is expected to enable writers to focus on idea generation to maximise writing production (Weigelt-Marom & Weintraub, 2018). Importantly, studies assessing handwriting in primary schooling show that handwriting automaticity takes several years to develop (Limpo & Alves, 2020). The data from our study suggests that students are receiving little handwriting and typing instruction in Australian primary classrooms. Given the unique and fundamental role that these transcriptions skills play in writing development, these findings are of concern.

As posited in the WWC model of writing (Graham, 2018), writing is shaped by the context in which writing development and instruction takes place, and shaped by the ‘affordances, constraints, or both, within the writing community” (p. 272). The importance of contextual variables potentially impacting writing instruction is illustrated by a comment made by two of the teachers in the present study, in the last open-ended section:

*“The curriculum is just too tight, teaching things like typing (and even handwriting) is hardly feasible”* and “*There needs to be a continued support and recognition for the importance of students being able to write—plan, draft, edit and present.”* Future research, including classroom observation studies, should seek to verify our study’s findings and attempt to identify factors that may influence the little time devoted to teaching these transcription skills and process writing skills in Australian primary classrooms.

### Extending writing to the home environment

We examined teachers’ practices to extend writing to the home environment. As predicted, the teachers in our study did not report making strong connections between home and school to support children’s writing. From the 20 practices assessed, home-school strategies were the least frequently reported strategies, with the majority of teachers reporting never asking students to write at home with parental assistance (54.5%). Moreover, most teachers indicated that they communicated with parents about their children’s writing performance (72%) or asked parents to read something their children write at school (77%) only once a year or never. Our findings replicate Cutler and Graham’s (2008) previous reports and are of concern when considering research showcasing the importance of home writing practices in supporting children’s writing development (Camacho & Alves, 2017; Saint-Laurent & Giasson, 2005). For example, when examining the impact of a program for Grade 1 students and their parents that included parental support for writing activities, Saint-Laurent and Giasson (2005) found a positive effect on students’ writing performance, including on spelling, vocabulary, and text length. In an intervention designed to promote parental involvement in writing carried out with Year 2 students and parents, Camacho and Alves (2017) also found a positive impact of parental involvement on children’s writing skills, including handwriting fluency, dictated spelling, text length and writing quality.

Emerging evidence on home writing practices during the pandemic suggest that the frequency and nature of writing activities in the home can vary significantly, and it is associated with factors such as the number of children in the home and the level of education of parents/guardians (López-Escribano et al., 2021). Despite data suggesting that strengthening home/school connections in support of home writing practices could be a fruitful strategy, our findings suggest that Australian families may be an underutilised resource in promoting children’s writing skills.

### Predictors of teachers’ reported writing practices: preparation and self-efficacy for teaching writing

The nature and frequency of writing instruction are related to teachers’ preparation and self-efficacy to teach writing (e.g., Banales et al., 2020; Gilbert & Graham, 2010). Aligned with reports from Chilean, English, and Portuguese educational contexts (i.e., Banales et al., 2020; Dockrell et al., 2016; Veiga-Simao et al., 2016), the majority of primary teachers participating in our study indicated having received adequate (42%) and very good (28%) pre-service preparation to teach writing. About 30% of teachers, however, believed their undergraduate preparation was poor or inadequate. Replicating results from previous studies examining writing instruction in England, Chile, Taiwan, Portugal, and New Zeeland (i.e., Dockrell et al., 2016; Banales et al., 2020; Hsiang et al., 2020; Veiga-Simao et al., 2016; Parr & Jesson, 2016), primary teachers participating in our study reported feeling overall confident in teaching writing. Of note is that nearly 47% of the teachers in our study reported feeling less confident in adjusting their practices to respond to students’ individual writing needs.

We examined if preparation and self-efficacy to teach writing predicted how teachers reported they taught writing, controlling for teacher and class variables (i.e., gender, years spent teaching, highest educational level, grade currently taught, and COVID-19 impact). Results indicated that both variables statistically accounted for variability in three of the four writing instruction factors we examined, namely evidence-based practices to promote effective writing, teaching foundational skills, and teaching process skills. Similar findings have been reported in international research, including in US, Chilean, Portuguese, Dutch, Norway, and Chinese primary educational contexts (i.e., Gilbert & Graham, 2010; Banales et al., 2020; Veiga-Simao et al., 2016; Rietdik et al., 2018; Graham et al., 2020; Hsiang et al., 2020). Our study further replicates findings from Banales and colleagues where preparation and self-efficacy for teaching writing accounted for variability in teachers’ reported practices even when teachers’ reported preparation and self-efficacy results were high. Hence, our findings offer additional support to the contention that self-efficacy and preparation for teaching writing are the two most consistent predictors of teachers’ reported writing instruction practices (Banales et al., 2020; Graham, 2019).

This is the first study, to our knowledge, examining the predictive value of self-efficacy and preparation to teach writing to develop teaching strategies aiming to extend writing to the home environment. When examining writing instruction in grades 1 through 3 in Taiwan, Hsiang and colleagues (2020) asked teachers to report on instructional activities they developed to provide extra writing assistance, including students writing at home with parental assistance; students reading their writing to parents; and teacher/parent communication. Their findings indicated that most teachers promoted extra writing assistance practices twice a year only and that teachers who felt more efficacious in teaching writing were more likely to provide extra writing assistance. As opposed to Hsian and colleagues’ survey (2020), the current survey included one factor identified as teaching practices to extend writing to the home environment, focusing more specifically on home and school connections.

Interestingly, our findings indicated that only self-efficacy for teaching writing made a unique and significant contribution to predicting school-home connections for writing. This finding is consistent with empirically validated associations between primary teachers’ self-efficacy and family involvement in schooling (Garcia, 2004; Hoover-Dempsey et al., 1987). For example, in her study of 110 American teachers, Garcia (2004) identified that teachers who felt more efficacious encouraged family involvement using a wider range of strategies when compared to those who felt less efficacious. Though the findings of our survey require further investigation, they suggest that efforts to enhance pre-service and in-service teacher efficacy on strategies that promote family involvement in writing may be warranted. We see this issue as a promising area of further scrutiny.

### Limitations and future research

This study has several limitations that should be considered and potentially addressed in future research. First, the relatively small sample size spanning teachers across Australian states and territories requires that caution must be applied in interpreting our findings. In Australia, state and territory authorities are responsible for implementing national literacy standards in their schools, but different versions operate across states and territories to address each state and territory contextual factors (Wall, 2017). While our findings corroborate results from cross-sectional studies (Malpique et al., 2017, 2020) and the only state survey we were able to find assessing writing instruction in Australian primary classrooms (Wyatt-Smith et al., 2018), we could not compare writing practices between states and territories due to the small sample size. Future research examining these differences is clearly needed to provide a more contextualised and comprehensive picture of writing instruction in Australian classrooms.

In our study, teachers were asked to report on typical writing practices and their self-efficacy for teaching writing before the impact of COVID-19. While similar national surveys have followed a retrospective approach to examine teachers’ reported practices instead of asking teachers to report on the practices being implemented at the time of taking the survey (e.g., Veiga Simao et al., 2016), such approach may have impacted teachers’ responses. Moreover, as our findings were collected via self-reports, data must be viewed cautiously as we did not actually assess the actual use of the specific writing practices in Australian primary classrooms. Another limitation of the current study was that teachers were only asked to report on their pre-service preparation for teaching writing. Future studies, including observational studies, must be developed to replicate and confirm our findings. Nevertheless, this is, to our knowledge, the first national study examining writing instruction in Australian primary education, providing additional information on the teaching of writing across the globe.

## Conclusion

The findings from our study, while consistent with previous research examining writing instruction in other countries, raise several concerns regarding the teaching of writing in Australian primary classrooms. First, similar to previous research, the participating teachers in this study reported applying different evidence-based practices to teach writing. However, most of the assessed practices were reportedly applied sparingly, with teachers stating that they typically allocated more time to teaching spelling skills than teaching handwriting, typing, and process writing skills, such as planning and revising. Considering the robust body of empirical evidence showcasing the importance of providing more recurrent opportunities for the development of such writing skills in primary education, efforts must be made to support teachers in addressing this need, including professional development initiatives at the national and state levels.

Replicating findings from studies across the globe, our study confirms the role of preparation and self-efficacy for teaching writing in teachers’ overall instructional practices for writing. Findings from our study, however, expand knowledge in this field, with results suggesting that self-efficacy for teaching writing may play a unique role in the way teachers develop opportunities to extend writing to the home environment. While we did not seek to establish relationships between any of the teachers’ practices here assessed and their students’ writing achievements, parental involvement in writing has been associated with better writing outcomes in primary education (Alston-Abel & Berninger, 2018). Thus, the development of teacher education programs that provide information and strategies to establish effective school-home connections for writing are clearly needed. Overall, more effective teacher education programs for writing need to be designed and implemented, as illustrated by a comment made by one of the teachers when responding to our survey: *While I have answered some of the questions in this survey negatively, I am trying to get better at teaching writing. I want to love it but I'm not sure exactly where to turn for help.* Overall, the findings from this study help provide information about writing instruction in Australian primary classrooms to inform evidence-based teacher education programs and initiatives to support teachers in the challenging and demanding task of promoting effective writing development in the primary years of schooling.
